# Characterization of SLCO5A1/OATP5A1, a Solute Carrier Transport Protein with Non-Classical Function

**DOI:** 10.1371/journal.pone.0083257

**Published:** 2013-12-20

**Authors:** Katrin Sebastian, Silvia Detro-Dassen, Natalie Rinis, Dirk Fahrenkamp, Gerhard Müller-Newen, Hans F. Merk, Günther Schmalzing, Gabriele Zwadlo-Klarwasser, Jens Malte Baron

**Affiliations:** 1 Department of Dermatology and Allergology, University Hospital RWTH Aachen, Aachen, Germany; 2 Institute of Pharmacology and Toxicology, Medical Faculty of the RWTH Aachen University, Aachen, Germany; 3 Institute of Biochemistry and Molecular Biology, Medical Faculty of the RWTH Aachen University, Aachen, Germany; 4 Interdisciplinary Center for Clinical Research (IZKF), Medical Faculty of the RWTH Aachen University, Aachen, Germany; Biological Research Centre of the Hungarian Academy of Sciences, Hungary

## Abstract

Organic anion transporting polypeptides (OATP/SLCO) have been identified to mediate the uptake of a broad range of mainly amphipathic molecules. Human OATP5A1 was found to be expressed in the epithelium of many cancerous and non-cancerous tissues throughout the body but protein characterization and functional analysis have not yet been performed. This study focused on the biochemical characterization of OATP5A1 using *Xenopus laevis* oocytes and Flp-In T-REx-HeLa cells providing evidence regarding a possible OATP5A1 function. SLCO5A1 is highly expressed in mature dendritic cells compared to immature dendritic cells (∼6.5-fold) and SLCO5A1 expression correlates with the differentiation status of primary blood cells. A core- and complex- N-glycosylated polypeptide monomer of ∼105 kDa and ∼130 kDa could be localized in intracellular membranes and on the plasma membrane, respectively. Inducible expression of SLCO5A1 in HeLa cells led to an inhibitory effect of ∼20% after 96 h on cell proliferation. Gene expression profiling with these cells identified immunologically relevant genes (e.g. CCL20) and genes implicated in developmental processes (e.g. TGM2). A single nucleotide polymorphism leading to the exchange of amino acid 33 (L→F) revealed no differences regarding protein expression and function. In conclusion, we provide evidence that OATP5A1 might be a non-classical OATP family member which is involved in biological processes that require the reorganization of the cell shape, such as differentiation and migration.

## Introduction

The organic anion transporting polypeptide (OATP) family belongs to the gene superfamily of solute carriers (SLC) and is classified within as gene family SLC21A (SLCO). Eleven members of the OATP family have been identified in human tissues, encoded by genes named SLCO (solute carrier organic anion transporter) (Hagenbuch & Meier, 2004). Mammalian OATPs are classified based on amino acid sequence homology and are grouped in 6 families, OATP1 to OATP6 [1]. Interestingly, the OATP family members are poorly conserved evolutionarily and orthologues for human OATPs may not exist in rodents [2].

The predicted secondary structure of the OATPs consists of twelve transmembrane domains yielding six extracellular and five intracellular loops with both N-and C-termini facing the cytosol [1]. A common transport mechanism has been proposed for all OATPs, in which substrates are translocated through a central, positively charged pore in a rocker-switch-type mechanism [3]. However, it is unclear whether this transport mode involves the coupled movement of another solute across the membrane or if it occurs by facilitated diffusion through the putative central pore [4].

OATPs form a family of influx transmembrane transporters expressed in various tissues, including the liver, the kidney, and the brain. They mediate the sodium-independent transport of a diverse range of mainly amphipathic organic compounds with molecular weights of more than 300 kDa, including bile acids, steroid conjugates, thyroid hormones, anionic peptides, numerous clinically important drugs [5], and other xenobiotic substances [6]. The skin, known for its metabolizing abilities [7]–[10], also represents a tissue for OATP-mediated transport. We have shown that OATP2B1 (formerly called OATP-B), OATP3A1 (OATP-D) and OATP4A1 (OATP-E) are constitutively expressed in normal human epidermal keratinoytes (NHEKs) and that the uptake of estradiol-17β-D-glucoronide and estrone-3-sulfate is inhibited by taurocholate in NHEKs [11]. Numerous sequence variations such as single nucleotide polymorphisms (SNPs) have been identified in SLCO genes [5], [12], [13]. Several of these SNPs have been linked to altered distribution of chemotherapeutic drugs and consequently increased adverse effects, confirming the importance of OATPs in the transport of drugs [14].

The OATP5 family consists of the sub-family OATP5A where OATP5A1 represents the only member in human, rat and mouse [15]. The putative OATP5A1 polypeptide contains 848 amino acids corresponding to a calculated molecular mass of 92 kDa. According to the NCBI-Gene website, alternative splicing results in transcript variants (793 aa/86 kDa, 687 aa/75 kDa). According to UniprotKB (ref. seq. Q9H2Y9), a natural variation with a SNP leading to the exchange of amino acid 33 (L→F) was identified (rs3750266). Among the SLCO family members, SLCO5A1 is the only gene which is located on chromosome 8 (8q13.3). High mRNA levels were detected in the brain, heart, skeletal muscle, and ovary [16]. SLCO5A1 was observed in human bone tumors, in prostate cancer [17] and in normal and cancerous breast tissue [18]. SLCO5A1 was also found in drug-resistant small cell lung cancer (SCLC) cells [19], primary liver cancer and liver metastases from colon tumors [20]. OATP5A1 protein is only described by two publications, which analyzed OATP5A1 expression by immunohistochemistry and immunofluorescence in paraffin-embedded tissue samples. OATP5A1 was found at the plasma membrane of epithelial cells of the lactiferous ducts in normal breast tissue and at the plasma membrane and in the cytoplasm of malignant breast tumor specimens [21]. OATP5A1 was also observed on the plasma membrane and in the cytosol of hepatic tumor cells, and additionally in various cytokeratin 19 positive bile ducts [20]. An expression profile of OATP5A1 protein can be viewed at ‘http://www.proteinatlas.org/tissue_profile.php?antibody_id=25062’, with the strongest expression in cortical cells of the adrenal gland and follicle cells of the ovary, though the specificity of the used antibody needs to be determined.

Although the expression profile of SLCO5A1 is well described, so far, the OATP5A1 polypeptide cell/tissue localization and function has not been further characterized and substrates have not yet been determined. In the present study, we analyzed the biochemical properties of human OATP5A1 recombinantly expressed in *Xenopus (X.) laevis* oocytes and genetically modified HeLa cells (‘Flp-In T-REx-HeLa’) and provided evidence regarding a possible OATP5A1 function. In this study both mRNA and protein were named SLCO5A1 for consistency.

## Materials and Methods

### Ethics Statement

For cell culture experiments with primary blood cells, buffy coats from anonymous healthy donors were purchased from the Department of Transfusion Medicine of the University Hospital RWTH Aachen (Germany) in accordance with the Aachen Ethics Committee.

### Isolation and cultivation of human primary blood cells

Human peripheral blood mononuclear cells (PBMCs) from four healthy donors were separated from buffy coats (Department of Transfusion Medicine, University Hospital RWTH Aachen, Germany) over a Ficoll-Paque gradient (Amersham Pharmacia Biotech, Uppsala, Sweden). Leukocytes positive for CD3, CD7, CD16, CD19, CD56, CD123 and Glycophorin A were depleted using a negative monocyte isolation kit (Human Monocyte Isolation Kit II, Miltenyi Biotec, Bergisch Gladbach, Germany). The isolated monocytes (CD14-positive) were cultured in medium consisting of RPMI 1640 + GlutaMAX™-I (Invitrogen, Darmstadt, Germany) with 10% human serum. Monocytes were harvested on day one and macrophages on day six to seven. For the generation of dendritic cells (DC), monocytes were cultured in 6-well plates at a density of 1x10^6^ cells/ml in 3 ml RPMI 1640 + GlutaMAX™-I supplemented with 3% autologous plasma in the presence of 800 U/ml GM-CSF and 1000 U/ml IL-4 (R&D-Systems, Bühlmann, Basel, Switzerland) for six days. Every other day 1 ml culture medium was replaced by 1 ml fresh cytokine-containing medium according to the protocol of Jonuleit et al. (1997) [22]. On day six the immature dendritic cells (iDC) were harvested. The resulting DC phenotype was determined by flow cytometric analysis. At this time point, the cells displayed a phenotype characteristic for iDC, i.e., CD1a^high^, CD80^intermediate^, CD86^low^, CD83^negative^ and CD14^negative^. On day seven maturation of DC was induced with medium containing a cocktail of pro-inflammatory cytokines (IL-6, IL-1β, TNFα (R&D-Systems)) together with PGE_2_ (Sigma-Aldrich, St. Gallen, Switzerland) as described [23]. On day nine to ten CD83-positive cells were harvested.

### Cloning

For the generation of expression plasmids the Gateway® cloning system (Invitrogen, Darmstadt, Germany) was used. Insertion of short sequences encoding affinity tags or point mutations were achieved by using the QuikChange Site-Directed Mutagenesis Kit (Stratagene, La Jolla, CA, USA) according to the manufacturer's instruction.

The open reading frame (ORF) sequence for human SLCO5A1 (transcript variant 1; NCBI Reference Sequence: NM_030958.2) was amplified by PCR using cDNA isolated from mature dendritic cells with a primer pair containing recombinational cloning sites (attB) for Gateway® recombinational cloning (Table 1a). The SLCO5A1 coding sequence was subcloned into Gateway-compatible versions of the oocyte expression vector pNKS4 [24] and the mammalian inducible expression vector pcDNA™5/FRT/TO (Invitrogen, Darmstadt, Germany). DNA sequences were confirmed by sequencing and revealed a SNP on position 33 (L→F). According to UniprotKB (ref. seq. Q9H2Y9), this sequence corresponds to a natural variation (rs3750266). The SNP was modified to the wild-type (WT) sequence with mutagenic primers that incorporated a Bsu36I restriction site (Table 1b). Additionally, the codons for a hexahistidine (His) tag were introduced immediately 3′ or 5′ of the ATG start codon or the stop codon, respectively, to generate pNKS4-His-hSLCO5A1^WT/L33F^ and pNKS4-hSLCO5A1^WT/L33F^-His by using mutagenic primers that incorporated a silent NsiI or BsmAI restriction site (Table 1c, d). The hSLCO5A1^WT/L33F^ ORF in the pcDNA5/FRT/TO vector was extended with a sequence encoding either a C-terminal HA (haemagglutinin)-tag using mutagenic primers that incorporated a silent SacI restriction site (Table 1e) or YFP (yellow fluorescent protein). The YFP sequence was attached in two cloning-steps. In a first step, mutagenic primers were used which modified the stop codon and inserted a linker sequence and a NheI restriction site 5′ of the hSLCO5A1^WT/L33F^ sequence (Table 1f). In a second step, the modified plasmids and the vector pEYFP-N1 (Clontech/Takara Bio Europe, Saint-Germain-en-Laye, France) were cut with NheI and NotI (NEB, Frankfurt/Main, Germany). The appropriate fragments were isolated from an agarose gel using the QiaQuick Gel Extraction Kit according to the manufacturer's instruction (Qiagen, Hilden, Germany) and were ligated with T4 DNA ligase (NEB, Frankfurt/Main, Germany). All constructs were verified by restriction analysis and DNA sequencing.

**Table 1:** Primer pairs for cloning.

Numbering	Primer pair (forward/reverse)
a	5′-GGGGACAAGTTTGTACAAAAAAGCAGGCTGCCATGGACGAAGGCACT-3′
	5′-GGGGACCACTTTGTACAAGAAAGCTGGGTTCAGGAGGGCGGCTCCAA-3′
b	5′-GCGAGCCGGAGACCCTCAGGTCTAAGAGTTTACCGG-3′
	5′-CCGGTAAACTCTTAGACCTGAGGGTCTCCGGCTCGC-3′
c	5′-CCAGTGCCTTCGTCGTGATGGTGATGGTGATGCATGGCAGCCTGCTTTTTTG-3′
	5′-CAAAAAAGCAGGCTGCCATGCATCACCATCACCATCACGACGAAGGCACTGG-3′
d	5′-CAAGAAAGCTGGGTTCAGTGGTGGTGGTGGTGGTGAGACGGCGGCTCCAAGG-3′
	5′-CCTTGGAGCCGCCGTCTCATCACCATCACCATCACTGAACCCAGCTTTCTTG-3′
e	5′-GGAGCCGCCCTCCTACCCATACGATGTTCCAGATTACGCTTGAGCTCAGCTTTCTTGTACAAAG-3′
	5′-CTTTGTACAAGAAAGCTGAGCTCAAGCGTAATCTGGAACATCGTATGGGTAGGAGGGCGGCTCC-3′
f	5′-GGAGCCGCCCTCCGGCGGCGAGCAGAAGCTGGCTAGCCCCAGCTTTCTTGTAC-3′
	5′-GTACAAGAAAGCTGGGGCTAGCCAGCTTCTGCTCGCCGCCGGAGGGCGGCTCC-3′

### Expression of hSLCO5A1 in *X. laevis* oocytes

The constructs pNKS4-His-hSLCO5A1^WT/L33F^ and pNKS4-hSLCO5A1^WT/L33F^-His were linearized 3′ to a polyA tail with XhoI and purified using the Qiaquick Nucleotide Removal Kit according to the manufacturer's instructions (Qiagen, Hilden, Germany). Capped cRNAs were transcribed *in vitro* with SP6 RNA polymerase (Epicentre Biotechnologies, Madison, USA) and purified and quantified as described [25]. The integrity of the cRNAs was confirmed by agarose gel electrophoresis followed by ethidium bromide staining. Collagenase-defolliculated *X. laevis* oocytes (stage V or VI) were isolated and injected with 50 nl of cRNA (0.03–1 µg/μl) as described [25]. Oocytes were incubated at 19°C in sterile frog Ringer's solution (ORi: 90 mM NaCl, 1 mM KCl, 1 mM CaCl_2_, 1 mM MgCl_2_, and 10 mM HEPES; pH 7.4) supplemented with 50 mg/l of gentamycin.

### Biochemical characterization of *X. laevis* oocyte-expressed hSLCO5A1

The oocytes were metabolically labelled by overnight incubation with L-[^35^S]-methionine, chased for 24 h in L-[^35^S]-methionine-free medium and surface-labelled with the membrane-impermeable infrared dye IR800-NHS just prior to protein extraction as previously described [26], [27]. The His-tagged proteins were purified by affinity chromatography using nickel-nitrilo acetic acid (Ni-NTA) agarose (Qiagen, Hilden, Germany) and eluted with non-denaturing elution buffer consisting of 0.5% digitonin and 250 mM imidazol/HCl (pH 7.4).

Immediately after their purification, the proteins were resolved by blue native (BN)-PAGE in the presence of 0.02% (w/v) Coomassie blue G250 [28]. Where indicated, the samples were treated with 0.01–0.1% (w/v) SDS for 1 h at 37°C prior to BN-PAGE to test for the presence of non-covalent protein complexes that should dissociate into their protomers upon denaturation by SDS. After complete destaining in an acetonitrile mixture as previously described [27], the BN-PAGE gel was scanned wet in an Odyssey Scanner (LI-COR Biosciences, Bad Homburg, Germany). For the subsequent visualization of the ^35^S-labelled proteins, the BN-PAGE gels were dried, exposed to a phosphor screen, and scanned on a PhosphorImager (Storm 820, GE Healthcare, Freiburg, Germany).

For SDS-urea-PAGE (4–10% acrylamide), the samples were incubated for 10 min at 56°C in SDS-PAGE sample buffer in the absence or presence of 20 mM DTT, and electrophoresed in parallel with a blue-stained molecular-mass marker (Precision Plus Protein All Blue, BioRad, München, Germany). To investigate the N-glycosylation state of the proteins, the samples were incubated for 2 h in reducing SDS-PAGE sample buffer with either endoglycosidase H (EndoH) or PNGase F (NEB, Frankfurt/Main, Germany); the PNGase-treated sample was supplemented with 1% (w/v) Nonidet P40 to counteract the SDS-mediated inactivation of PNGaseF. After electrophoresis, the SDS-PAGE gels were scanned directly on an infrared fluorescence scanner (Odyssey, LI-COR Biosciences, Bad Homburg, Germany) to visualize the fluorescently-labelled plasma membrane-bound proteins. Subsequently, the gels were dried and exposed to a PhosphorImager screen for the detection of the ^35^S-incorporation as described above.

### Transport assay in *X. laevis* oocytes expressing hSLCO5A1

Tritium- labelled transport substrates were purchased from Amersham (GE Healthcare, Freiburg, Germany), Perkin Elmer (Waltham, Massachusetts, USA) (including [^14^C]sucrose (14.8–25.9 GBq/mmol)), or ARC (American Radiolabeled Chemicals, St. Louis, MO, USA) via Hartmann Analytic (Braunschweig, Germany).

Oocytes microinjected with 50 nl of one of the SLCO5A1 cRNAs (0.03–1 µg/μl) or Tris-HCl as a control were incubated at 19°C in ORi supplemented with 50 µg/ml of gentamycin for 48 h. Following prewashing in Ori, groups of 8–12 oocytes were incubated for 30 min at ambient temperature (20–22°C) in 100 µl of ORi containing 1 µCi/ml Tritium-labelled substrate and 0.04 µCi/ml [^14^C]sucrose. The [^14^C]sucrose served as an indicator for leaky cells [29]. After washing three times in ice-cold ORi, the oocytes were individually transferred into scintillation counting vials and lysed by overnight shaking in 250 µl of 5% SDS (Roth, Karlsruhe, Germany). A scintillation fluid compatible with aqueous samples was added and the radioactivity was counted in a Beckman LS6000SC scintillation counter (Beckman Coulter, Krefeld, Germany) with appropriate channel settings for simultaneous ^3^H and ^14^C counting.

### Flp-In T-REx-HeLa cell culture, transfection, and stable clone selection

Flp-In T-REx-HeLa cells (epithelial cervix carcinoma cells) (Invitrogen, Darmstadt, Germany) allow the tetracycline-inducible expression of a gene of interest from a specific genomic location (for more information see the Flp-In™ T-REx™ Core Kit Manual from Invitrogen). Stable SLCO5A1-expressing Flp-In T-REx-HeLa cells were generated using the FlpIn recombinase-mediated system kit (Invitrogen, Darmstadt, Germany), which permits the targeted integration of genes to the same locus in all transfected cells to provide a homogeneous level of gene expression. To this end, cells were co-transfected with the FlpIn expression vector pcDNA5/FRT/TO (mock) or with the same vector containing the wild-type (WT) or mutant (L^33^F) sequence for SLCO5A1, modified C-terminally with either the sequence for a HA epitope or a YFP-tag or left unmodified, together with the Flp-recombinase expression vector pOG44 (Invitrogen, Darmstadt, Germany). Individual clones were separated by monoclonal selection with 15 µg/ml blasticidin (Invitrogen, Darmstadt, Germany) and 100 µg/ml hygromycinB (PAA, Pasching, Austria). Cells were cultured in EMEM supplemented with 10% FCS (tetracycline/doxycycline-reduced) (Biochrom, Berlin, Germany). SLCO5A1-expression was induced by adding 1 µg/ml tetracycline (tet) (Sigma-Aldrich, St. Gallen, Switzerland) to the Flp-In T-REx-HeLa cells (hereinafter referred to as HeLa cells).

### Western blot analysis of hSLCO5A1-expressing HeLa cells

HeLa cells were lysed on ice using RIPA buffer containing 10 mM Tris/HCl pH 7.4; 0.1% SDS; 150 mM NaCl; 1% NP40; 1% sodium deoxycholate and 0.5% aprotinin supplemented with proteinase inhibitors (AppliChem, Darmstadt, Germany). To investigate the N-glycosylation state of the proteins, HA-tagged samples of the WT SLCO5A1 and its L^33^F mutant were treated with either EndoH or PNGaseF (see section ‘Biochemical characterization of *X. laevis* oocyte-expressed hSLCO5A1’). Proteins (SLCO5A1^WT/L33F^-HA/-YFP) were separated by SDS-PAGE and electrophoretically transferred to PVDF membranes by wet blotting. The blots were probed with either the HA.11 Clone 16B12 monoclonal mouse antibody (Covance, Brussels, Belgium) or the mouse anti-GFP monoclonal antibody (Rockland Immunochemicals, Gilbertsville, PA, USA). To normalize sample loading, blots were stripped and reprobed with monoclonal anti-tubulin antibody (Sigma-Aldrich, St. Gallen, Switzerland). A polyclonal rabbit anti-mouse IgG:HRP antibody (Dako, Hamburg, Germany) was used as the secondary antibody. Bands were visualized using enhanced chemiluminescence (GE Healthcare, Amersham, U.K.).

### Confocal Fluorescence Microscopy

HeLa cells stably expressing the YFP-tagged WT SLCO5A1 or the L^33^F mutant were seeded in 12-well plates on cover-slips. On day two, SLCO5A1 expression was induced by adding 1 µg/ml tet. After 24 h cells were fixed with 3.7% paraformaldehyde for 15 minutes, washed with 1× PBS containing 1 mM MgCl_2_ and 0.1 mM CaCl_2_ and quenched with this PBS solution containing 50 mM NH_4_Cl for 5 minutes in the dark. After washing, cover-slips were stained with DAPI (1∶5000 in 1× PBS) (AppliChem/Biochemica, Darmstadt, Germany) for 5 minutes in the dark. Cover-slips were washed again and were embedded in Fluorescent Mounting Medium (Dako, Hamburg, Germany). Fluorescence of YFP and DAPI was visualized by confocal laser microscopy on a Zeiss LSM710 (Jena, Germany). The images were processed with the software ZEN2009/2011 (Zeiss, Jena, Germany).

### Proliferation assay

Stable HeLa cell clones (5×10^4^ cells) were seeded in 6-well plates in triplicates. After two hours at 37°C and 5% CO_2_ cells were treated with 1 µg/ml tet or left untreated. For a period of four days, every 24 h the total cell number was determined using the Casy cell counting system (Innovatis/Roche, Mannheim, Germany).

### RNA isolation

Cells were homogenized using QIAshredder spin columns (Qiagen, Hilden, Germany). Total RNA was isolated using the RNeasy Mini Kit (Qiagen, Hilden, Germany) according to the manufacturer's instructions, including on column digestion of DNA with RNase-free DNase I. RNA was quantified by photometric measurement (Nanodrop Technologies, Wilmington, Delaware, USA). For exon expression arrays, RNA integrity has been proven by using the 2100 Bioanalyzer (Agilent Technologies, Palo Alto, CA, USA).

### Reverse-Transcription PCR

1 µg of total RNA was reverse transcribed using the High Capacity RNA-to-cDNA Master Mix (Applied Biosystems, Darmstadt, Germany) or the SuperScript® VILO™ MasterMix (Invitrogen, Darmstadt, Germany) according to the manufacturer's instruction.

### Quantitative *real-time* reverse transcriptase polymerase chain reaction (qRT-PCR)

TaqMan experiments were carried out on an ABI PRISM 7300 Sequence Detection System (Applied Biosystems, Darmstadt, Germany) using TaqMan gene expression assay products according to the manufacturer's recommendations. Human Primer/probe sets for the following genes were obtained from the Applied Biosystems Assays-On-Demand repository: SLCO5A1 (Hs00229597_m1), DSC3 (Hs00170032_m1), TGM2 (Hs00190278_m1), OSMR (Hs00384276_m1), CCL20 (Hs01011368_m1) and CYP1B1 (Hs00164383_m1). Assay-on-Demand products for GAPDH (Hs99999905_m1) and GUSB (Hs99999908_m1) were used as an internal reference to normalize the target transcripts. Results were analyzed with the 7300 System SDS Software (Applied Biosystems, Darmstadt, Germany).

### Analysis of gene expression using exon expression arrays

Mock-transfected HeLa cells and HeLa cells expressing the WT SLCO5A1 were both treated with 1 µg/ml tet for 24 h and then RNA of the samples was collected by centrifugation using the RNeasy Mini Kit (Qiagen, Hilden, Germany). The Ambion® WT Expression Kit (Ambion, Kaufungen, Germany) was used to generate purified sense-strand cDNA with incorporated dUTP according to the technical manual. Fragmentation and labeling was done using the Affymetrix Gene Chip® WT Terminal Labeling kit (Affymetrix, Santa Clara, CA, USA) according to the manufacturer's recommendations [30]. Each sample was hybridized to a GeneChip Human Exon 1.0 ST array for 16 h at 45°C. Expression values of each probe were determined and the mock sample was compared to the WT SLCO5A1 sample using the GeneSpring® GX 12.0 software (Agilent Technologies, Frankfurt/Main, Germany). Genes with a fold change of at least 2.0 were analyzed.

### Statistical Analysis

Test of significance was performed by Student's *t* test using Sigma Plot Version 11.0 software (Systat Software GmbH, Erkrath, Germany). *p<0.05; **p<0.005.

## Results

### Human SLCO5A1 is differentially expressed in primary blood cells

**Figure 1:**
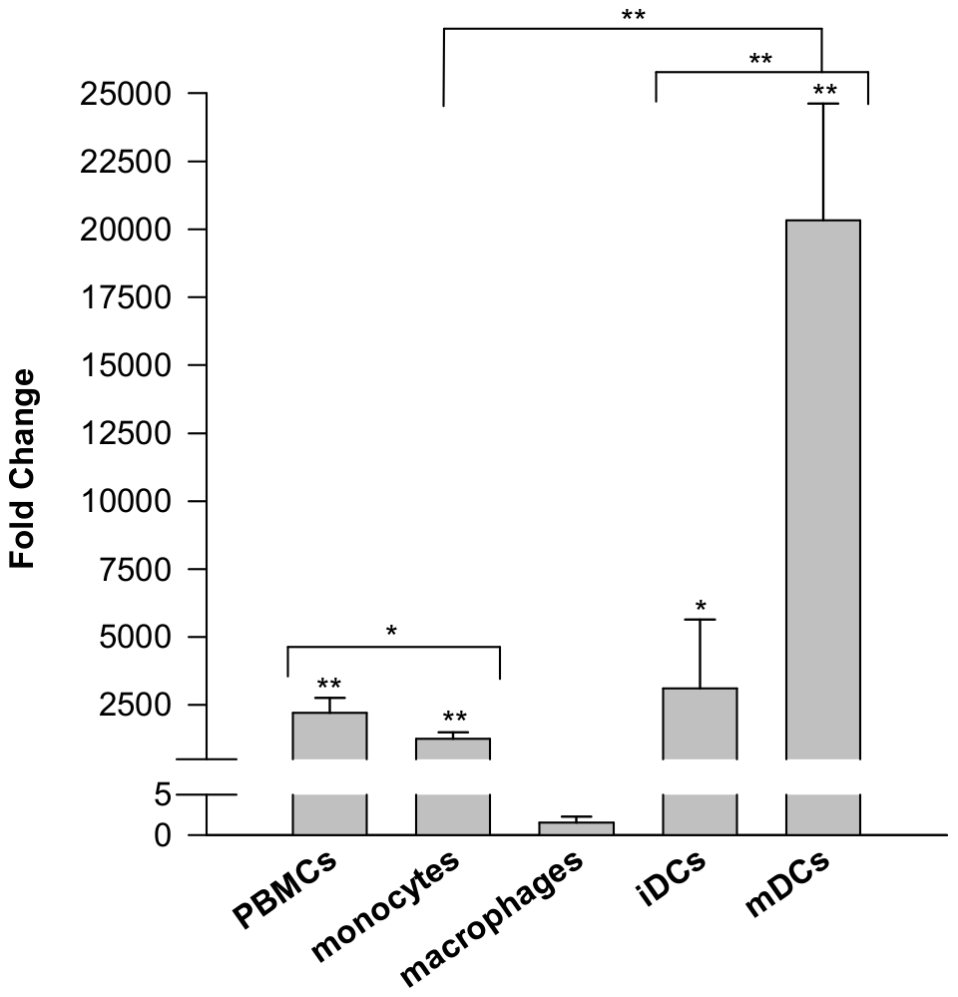
Human SLCO5A1 mRNA expression in primary blood cells. Total SLCO5A1 RNA expression, measured by qRT-PCR, is shown as fold-change compared to the macrophage sample (Avg Ct∼34) and was normalized to GAPDH (glyceraldehyde-3-phosphate dehydrogenase) mRNA expression. Mean values with standard deviation of 4 blood donors are displayed. (PBMC: peripheral blood mononuclear cells, iDC: immature dendritic cells, mDC: mature dendritic cells). *p<0.05; **p<0.005.

Quantitative RT-PCR revealed a statistically significant expression of SLCO5A1 mRNA in peripheral blood mononuclear cells (PBMCs), monocytes, immature dendritic cells (iDCs) and mature dendritic cells (mDCs) as compared to macrophages, which only showed a very low mRNA expression of SLCO5A1 (Fig. 1). Interestingly, SLCO5A1 expression strongly increased during the differentiation from monocytes to mDCs but decreased during the differentiation from monocytes to macrophages. The expression of SLCO5A1 in PBMCs and monocytes did not differ significantly from the expression in iDCs.

### Core- and complex-N-glycosylated human WT and mutant SLCO5A1 proteins are expressed on the cell surface of *X. laevis* oocytes

**Figure 2:**
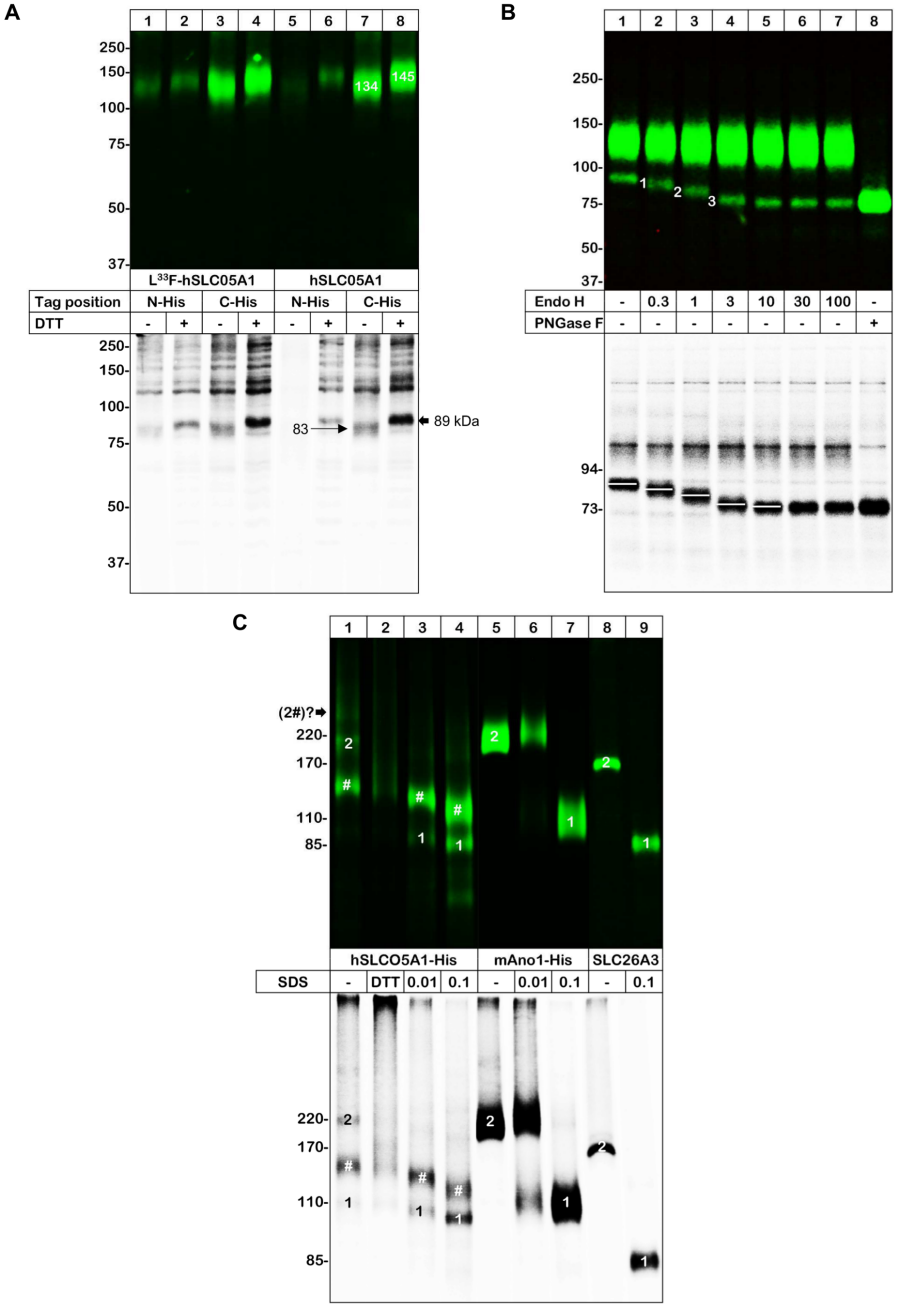
Biochemical characterization of *X. laevis* oocyte-expressed hSLCO5A1. *X. laevis* oocytes expressing the WT SLCO5A1 and its L^33^F mutant with a N- or C-terminal His-tag were [^35^S]methionine-labelled, chased, surface-labelled with the membrane-impermeable infrared (IR) dye 800-NHS and then extracted with digitonin. Proteins were purified by Ni-NTA chromatography, resolved by reducing SDS-urea-PAGE (A, B) or BN-PAGE (C), and visualized by phosphorimaging and Odyssey IR scanning to display the total (lower panel, black) and cell-surface (upper panel, green) pools of the proteins, respectively. A molecular-mass marker (All Blue Standard, BioRad) was electrophoresed in parallel. A) SDS-urea-PAGE analysis under non-reducing (−) and reducing conditions (+) by addition of 20 mM DTT. B) SDS-urea-PAGE analysis for the investigation of the glycosylation state of the C-terminal His-tagged WT SLCO5A1 protein. Samples were treated with either different concentrations of endoglycosidase H (EndoH) or with PNGase F or were left untreated. C) BN-PAGE analysis of the higher order structure of the C-terminal His-tagged WT SLCO5A1 protein. Where indicated, the samples were treated with 0.01–0.1% SDS to induce partial dissociation of the membrane transporter complexes. The proteins mAno1-His (lanes 5–7) and SLC26A3 (lanes 8, 9) served as dissociation control for the oligomeric protein structure. (2, homodimer of the high-mannose-glycosylated SLCO5A1 protein; 1, monomer of the high-mannose-glycosylated SLCO5A1 protein; #, monomer of the complex-glycosylated SLCO5A1 protein).

We expressed the wild-type (WT) SLCO5A1 and its L^33^F mutant with a N- or C-terminal His-tag in *X. laevis* oocytes and purified the metabolically ^35^S-labelled and surface-labelled SLCO5A1 proteins by one step non-denaturing affinity chromatography. To preserve the possible oligomeric state of SLCO5A1 we used digitonin for the solubilization, which behaved in our previous experiments as a very mild detergent for integral membrane proteins [26], [27], [31]. The purified SLCO5A1 proteins were analyzed in their SDS-denatured and non-denatured states by SDS-urea-PAGE and BN-PAGE, respectively. Independent of the position of the His-tag or the presence of the L^33^F mutation, the SDS-denatured [^35^S]methionine-labelled SLCO5A1 polypeptides (854 amino acids each including the His-tag, corresponding to a calculated protein core of 92 kDa) migrated in linear SDS-PAGE gels with apparent molecular masses of about 83 kDa and 89 kDa in the non-reduced and reduced form, respectively (Fig. 2A, lower panel). The 6 kDa larger mass of the reduced state can be assigned to the increase in the hydrodynamic radius that typically occurs when the intrasubunit disulfide bonds of polypeptides are cleaved. The plasma membrane-bound SLCO5A1 polypeptide was expressed predominantly in a form migrating at ∼134 kDa and ∼145 kDa in the non-reduced and reduced state, respectively (Fig. 2A, upper panel). In all the experiments, the C-terminally His-tagged versions of SLCO5A1 were more strongly expressed than the N-terminally His-tagged versions. No differences in the expression and migration patterns between the WT SLCO5A1 and the L^33^F mutant were observed.

Treatment with PNGase F, which removes all types of N-glycans [32], deglycosylated both the 89 kDa form and the 130 kDa form (corresponding to the 145 kDa form in Fig. 2A) of SLCO5A1 to the same protein core of 74 kDa (Fig. 2B, lane 8). In contrast, only the 89 kDa form could be deglycosylated with Endo H (Fig. 2B, lane 7), which removes glycans of the high-mannose type, but not of the complex-type [32]. This differential sensitivity to Endo H and PNGase F identifies the 89 kDa and the 130 kDa bands to represent the high-mannose and complex-glycosylated form of SLCO5A1, respectively. It has to be noted that the 89 kDa band was seen as a plasma membrane-bound protein in most of the experiments (Fig. 2B, upper panel) though not in the experiment displayed in Fig. 2A. The complex-glycosylated SLCO5A1 polypeptide is by far the most abundant SLCO5A1 species at the plasma membrane (Fig. 2B, upper panel). In the metabolically ^35^S-labelled form the complex-glycosylated SLCO5A1 is less well visible apparently because of its diffuse migration and lower abundance compared to the high-mannose-type SLCO5A1 (Fig. 2B, lower panel).

The experimentally observed mass of the N-glycan-free core SLCO5A1 protein of 74 kDa produced by PNGase F treatment is 20% lower than the 92 kDa expected from the deduced amino acid sequence. Because the SLCO5A1 polypeptides migrated at the same molecular masses irrespective of whether the purification was achieved by an N- or C-terminal His-tag, we exclude proteolytical cleavage as a possible cause and attribute the lower-than-expected mass to an anomalous fast electrophoretic mobility. A similar phenomenon has repeatedly been observed with highly hydrophobic membrane proteins including SLC proteins [33], [34].

The primary sequence of SLCO5A1 shows a total of nine N-glycosylation motifs of the type N-X-S/T-X (where X can be any amino acid except proline). Of these nine motifs, six are located in N-glycosylatable positions in the predicted extracellular loops two and five in distances of at least 12 residues to the membrane according to the membrane topology program MEMSAT 3. For a rough estimate how many of these motifs may be used, we deglycosylated the SLCO5A1 polypeptide with increasing concentrations of Endo H. We observed a reduction of the mass of the high-mannose SLCO5A1 from 89 kDa in three steps of 3.1 kDa, 5.7 kDa and 6.3 kDa (numbered 1–3 in Fig. 2B, lanes 2–7). These findings suggest that the SLCO5A1 polypeptide carries at least three N-glycans; 5-6 N-glycans are predicted when an average mass of 3 kDa per high-mannose-type N-glycan is considered.

To assess whether the SLCO5A1 polypeptide assembles into stable higher order structures, we resolved the non-denatured and partially SDS-denatured SLCO5A1 protein by BN-PAGE (Fig. 2C). As membrane-bound marker proteins covering the 85–220 kDa range, we used the DIDS-sensitive anion exchanger SLC26A3 (solute carrier family 26, subfamily A, member 3) and the Ca^2+^-activated chloride channel mAno1 (mouse anoctamin 1-His) that we have previously identified as homodimeric proteins [27], [34]. The 220 kDa mAno1 homodimer and the 170 kDa SLC26A3 homodimer dissociated in the presence of 0.1% SDS almost completely into protomers of 110 kDa and 85 kDa, respectively (Fig. 2C, lanes 7 and 9), as previously described [27], [34]. Based on the masses of these integral membrane proteins, the two ^35^S-labelled non-denatured SLCO5A1 proteins were calculated to migrate with apparent masses of 220 kDa and 147 kDa (Fig. 2C, lower panel, lane 1). In addition, a significant amount of aggregates was visible at the top of the BN-PAGE gel. Incubation with 0.01% SDS resulted in a disappearance of the 220 kDa band including most of the aggregates and the appearance of a new band of 106 kDa, suggesting that the 220 kDa band represents the SLCO5A1 homodimer. Furthermore, 0.01% of SDS slightly accelerated the migration of the 147 kDa band to an apparent mass of 136 kDa (Fig. 2C, lower panel, lane 3). Increasing the SDS concentration to 0.1% further accelerated the migration of the 136 kDa and the 106 kDa bands, but did not lead to the appearance of a novel band (Fig. 2C, lower panel, lane 4) that may be interpreted to be a protomer of the 146 kDa band (Fig. 2C, lane 1). Taking also the results of SDS-PAGE into account (Fig. 2A and B), we conclude that the two bands seen in the BN-PAGE gel after SDS treatment (Fig. 2C, lane 3, 4) represent the monomeric forms of the high-mannose and complex-glycosylated SLCO5A1 proteins. The two non-denatured proteins seen without prior SDS treatment in the BN-PAGE gel (Fig. 2C, lane 1) most likely represent the 220 kDa homodimer of the high-mannose-glycosylated SLCO5A1 (equal to twice the mass of the 106 kDa protomer) and the 147 kDa monomer of the complex-glycosylated SLCO5A1. According to this interpretation, the plasma membrane contains predominantly the complex-glycosylated monomeric form of the SLCO5A1, which migrates at ∼147 kDa in the absence of SDS and somewhat faster in the presence of SDS. In addition, the plasma membrane contains the 220 kDa homodimer of the high-mannose-glycosylated SLCO5A1. A further weak band migrating with a mass larger than 220 kDa may represent the homodimer of the complex-glycosylated SLCO5A1 with an expected mass of ∼300 kDa.

### Human WT and mutant SLCO5A1 N-glycosylated proteins are expressed in intracellular membranes and on the plasma membrane of HeLa cells

For the biochemical characterization of the SLCO5A1 polypeptide in mammalian cells, we stably expressed the C-terminally HA- or YFP-tagged WT SLCO5A1 and its L^33^F mutant in HeLa cells due to the lack of a commercially available suitable antibody against the SLCO5A1 protein.

**Figure 3:**
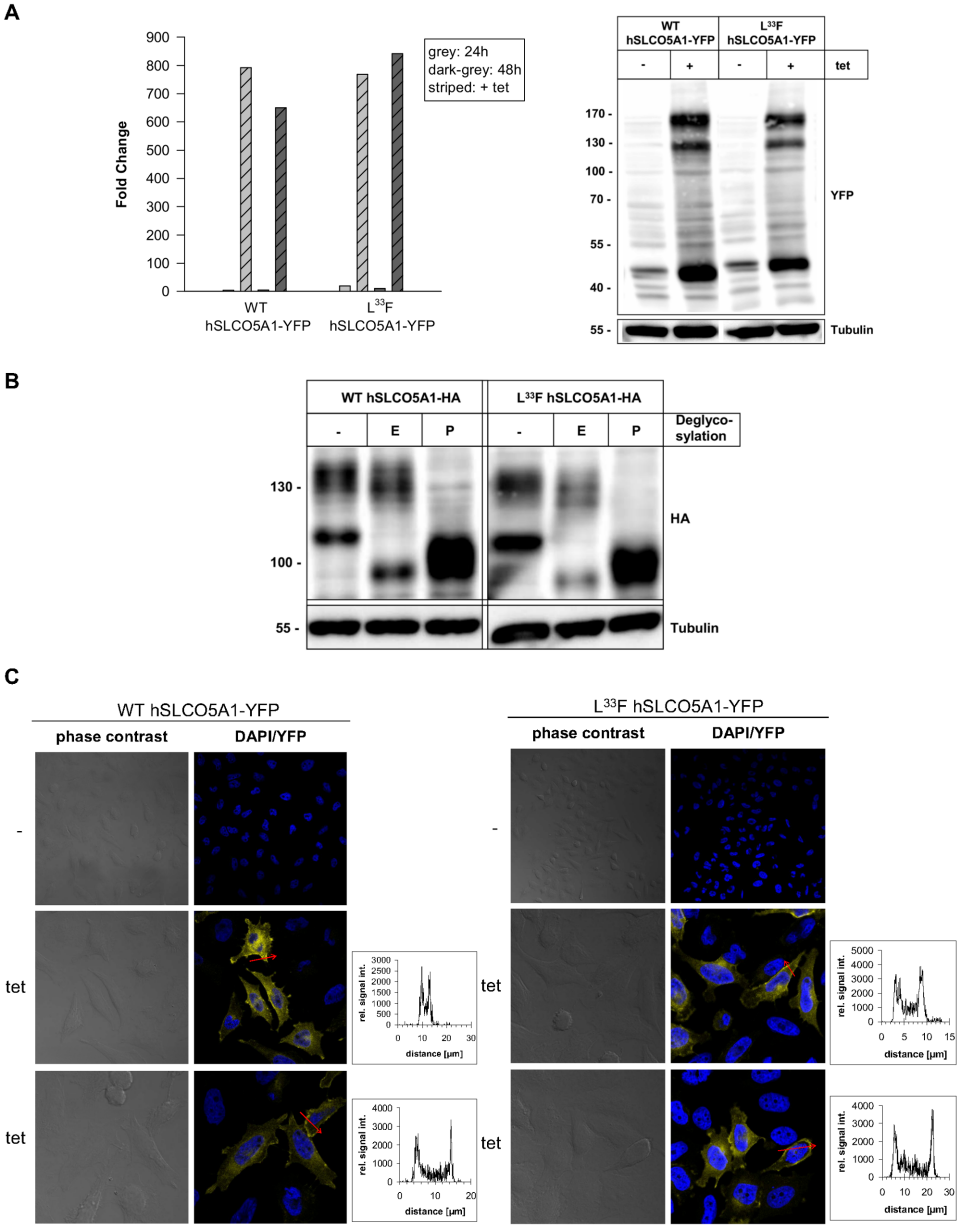
Inducible expression of hSLCO5A1 in HeLa cells. A) RNA and protein expression of the YFP-tagged WT SLCO5A1 and its L^33^F mutant. mRNA expression was measured after 24 h and 48 h treatment with 1 µg/ml tetracycline. As a basal mRNA expression control cells were left untreated. SLCO5A1 mRNA, measured by TaqMan qRT-PCR, was normalized to GAPDH mRNA expression. The relative RNA levels are presented as fold-change compared to SLCO5A1 expression in untransfected HeLa cells ( = 1). SLCO5A1 protein expression after treatment with 1 µg/ml tetracycline for 48 h was determined by western blot analysis. Tubulin served as loading control. B) Western blot analysis of the deglycosylated HA-tagged WT and mutant (L^33^F) SLCO5A1 protein. Protein expression was induced with tetracycline for 48 h. The proteins were deglycosylated with either endoglycosidase H (EndoH) (E) or PNGase F (P). Tubulin served as loading control. C) Protein expression of the YFP-tagged WT SLCO5A1 or its L^33^F mutant after induction with 1 µg/ml tetracycline for 24 h was analyzed by confocal fluorescence microscopy (blue: DAPI; yellow: YFP). The diagrams represent YFP fluorescence intensities along the length of the red arrows (x-axis: distance [μm]; y-axis: relative signal intensity).

Independent of the L^33^F mutation, the mRNA of the YFP-tagged SLCO5A1 was expressed in HeLa cells after induction with tetracycline (tet) in nearly the same amount (Fig. 3A). Both the YFP-tagged WT and the L^33^F mutant polypeptide were detected on an immunoblot as a double band of approximately 120 kDa and 160 kDa (Fig. 3A). A further protein band was visible at approximately 50 kDa, which did not appear using the HA-tagged SLCO5A1 protein samples. Endogenous expression of the HA- or YFP-tagged SLCO5A1 protein could not be observed (Fig. 3A, B).

The HA-tagged SLCO5A1 polypeptide was detected on an immunoblot as a double band of approximately 105 kDa and 130 kDa (Fig. 3B). The small HA-tag with a molecular mass of approximately 1 kDa has only a minor influence on SLCO5A1 protein size. Subtracting the YFP-tag of 27 kDa, the polypeptide sizes observed with HA- or YFP-tagged SLCO5A1 are quite similar. Treatment with PNGase F deglycosylated both the 105 kDa form and the 130 kDa form of SLCO5A1 to the same protein core of 92 kDa (corresponding to the calculated protein core) (Fig. 3B). In contrast, only the 105 kDa form could be deglycosylated with Endo H (Fig. 3B). This differential sensitivity to Endo H and PNGase F identifies the 105 kDa and the 130 kDa bands to represent the high-mannose and complex-glycosylated form of SLCO5A1, respectively (as observed also in *X. laevis* oocytes (Fig. 2B)). No differences in the N-glycosylation pattern between the WT SLCO5A1 and its L^33^F mutant were observed.

To localize the SLCO5A1 protein in the HeLa cell, YFP-tagged WT SLCO5A1 and the L^33^F mutant were determined by confocal fluorescence microscopy (Fig. 3C). The SLCO5A1 polypeptide was widely expressed throughout the cell and a strong expression was seen around the nucleus. Apart from the expression of the SLCO5A1 protein at intracellular membrane compartments, the protein was also found on the plasma membrane. It was noticeable that a certain amount of plasma membrane – expressed SLCO5A1-YFP seemed to be located at the cell extensions of HeLa cells. Furthermore, we observed that some of the HeLa cells seemed to express the SLCO5A1 protein in a lower amount or even not at all (∼ 35%). Possibly, the YFP-tagged SLCO5A1 protein expression may vary with the cell-cycle. No significant differences in the strength of expression and in the localization of the SLCO5A1 protein between the WT and its L^33^F mutant were found.

### Human WT and mutant SLCO5A1 proteins do not transport known OATP substrates

The SLCO5A1 protein was found to be expressed on the plasma membrane of *X. laevis* oocytes (Fig. 2A). To study whether the human SLCO5A1 protein shares transport activity across the plasma membrane with other OATP family members, SLCO5A1-expressing oocytes were incubated with a series of known OATP substrates that were available in tritiated form (Table 2). SLCO5A1-non-expressing oocytes served as negative controls. Notably, none of the substrates tested were found to be transported neither by the WT SLCO5A1 nor by the L^33^F mutant.

**Table 2:** Transport assay of the hSLCO5A1 protein with Tritium-labelled substrates in *X. laevis* oocytes.

Substrate [^3^H]	Specific activity	WT hSLCO5A1	L^33^F hSLCO5A1	control
Arachidonic acid	7.99 TBq/mmol	1074±133	1091±18	1439±168
Prostaglandin E_2_	6.73 TBq/mmol	ND	261±46	225±21
Estrone-3-sulfate	1.61 TBq/mmol	ND	118±55	89±51
Estradiol-17β-D-glucoronide	1.11–2.22 TBq/mmol	157±6	174±34	143±15
Dehydroepiandrosterone-3-	19.5 mCi/mmol	102±17	101±10	82±19
sulfate (DHEAS)				
[D-Penicillamine^2,5^]-	1.11–2.22 TBq/mmol	ND	52±22	20±4
enkephalin (DPDPE)				
Ouabain	30.6 Ci/mmol	129±55	137±61	112±31
Taurocholic acid	1–5 Ci/mmol	ND	23±15	11±2
Benzylpenicillin	25 Ci/mmol	249±59	272±66	319±41
Methotrexate	15 Ci/mmol	ND	95±48	62±11
Digoxin	20 Ci/mmol	ND	32±17	25±7
Leukotriene C_4_	3.7–8.88 TBq/mmol	ND	189±9	159±12

*X. laevis* oocytes (8–12 oocytes) were injected with the cRNA of the WT SLCO5A1 or its L^33^F mutant, or with the control (Tris-HCl). Oocytes were incubated with 1 µCi/ml Tritium-labelled substrate and 0.04 µCi/ml [^14^C]sucrose at room temperature for 30 minutes. [^14^C]sucrose served as internal leakage control. Radioactivity was measured using a Beckman scintillation counter. Mean CPM (counts per minute) values with standard deviation are displayed.

### Tet-induced WT SLCO5A1 gene expression inhibits HeLa cell proliferation

**Figure 4:**
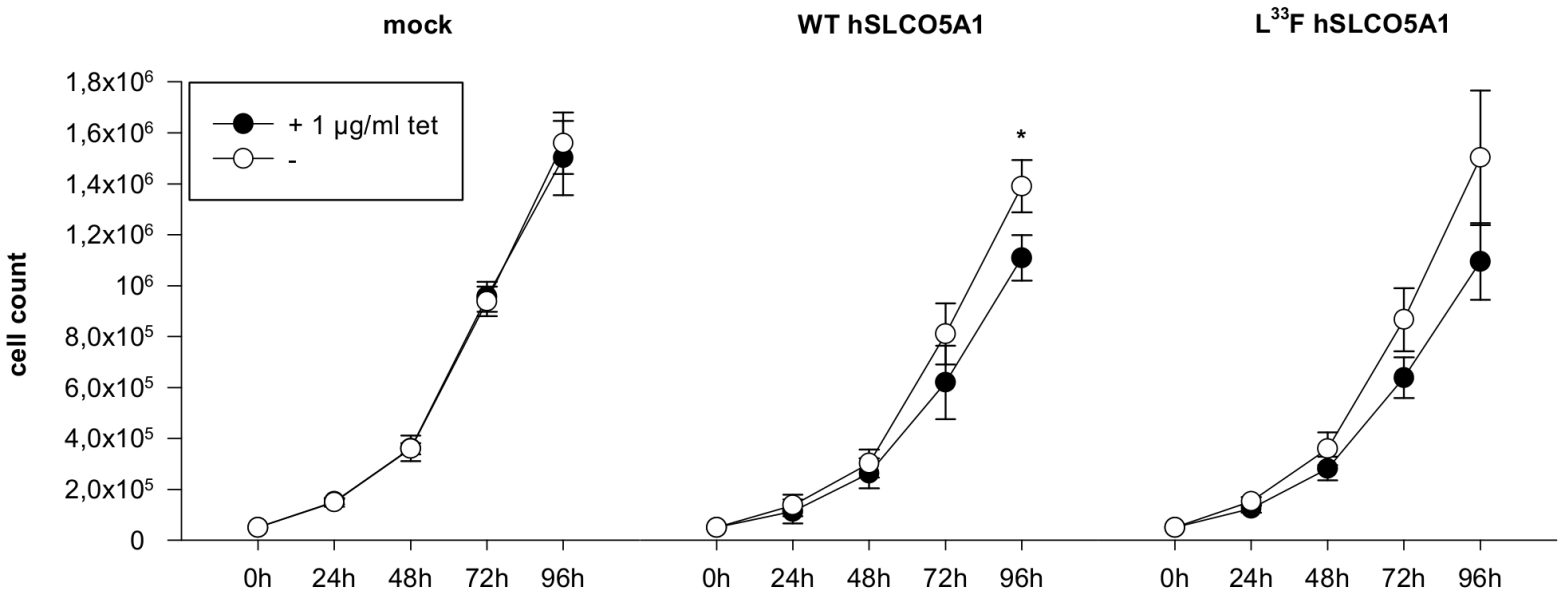
Proliferation assay of hSLCO5A1-expressing HeLa cells. The proliferation rate of stably transfected HeLa cells was measured by counting cells incubated in the presence or absence of tetracycline (1 µg/ml). At the indicated time points the total cell number was determined by using the Casy Counter System. Mean values with standard deviation of 3 biological replicates (*p = 0.023) are displayed.

The effect of SLCO5A1 on cell proliferation was studied in WT and mutant SLCO5A1-expressing HeLa cells and mock-transfected cells over a period of 96 h (Fig. 4). Interestingly, in WT cells, proliferation was significantly inhibited after 96 h induction of SLCO5A1 expression. Even though mutant SLCO5A1 expression did not result in a strong inhibition, the tendency of growth inhibition was the same as seen in the WT cells (see also figure S1).

### Gene expression profiling of Tet-induced SLCO5A1 expression in HeLa cells identifies genes involved in developmental processes

**Figure 5:**
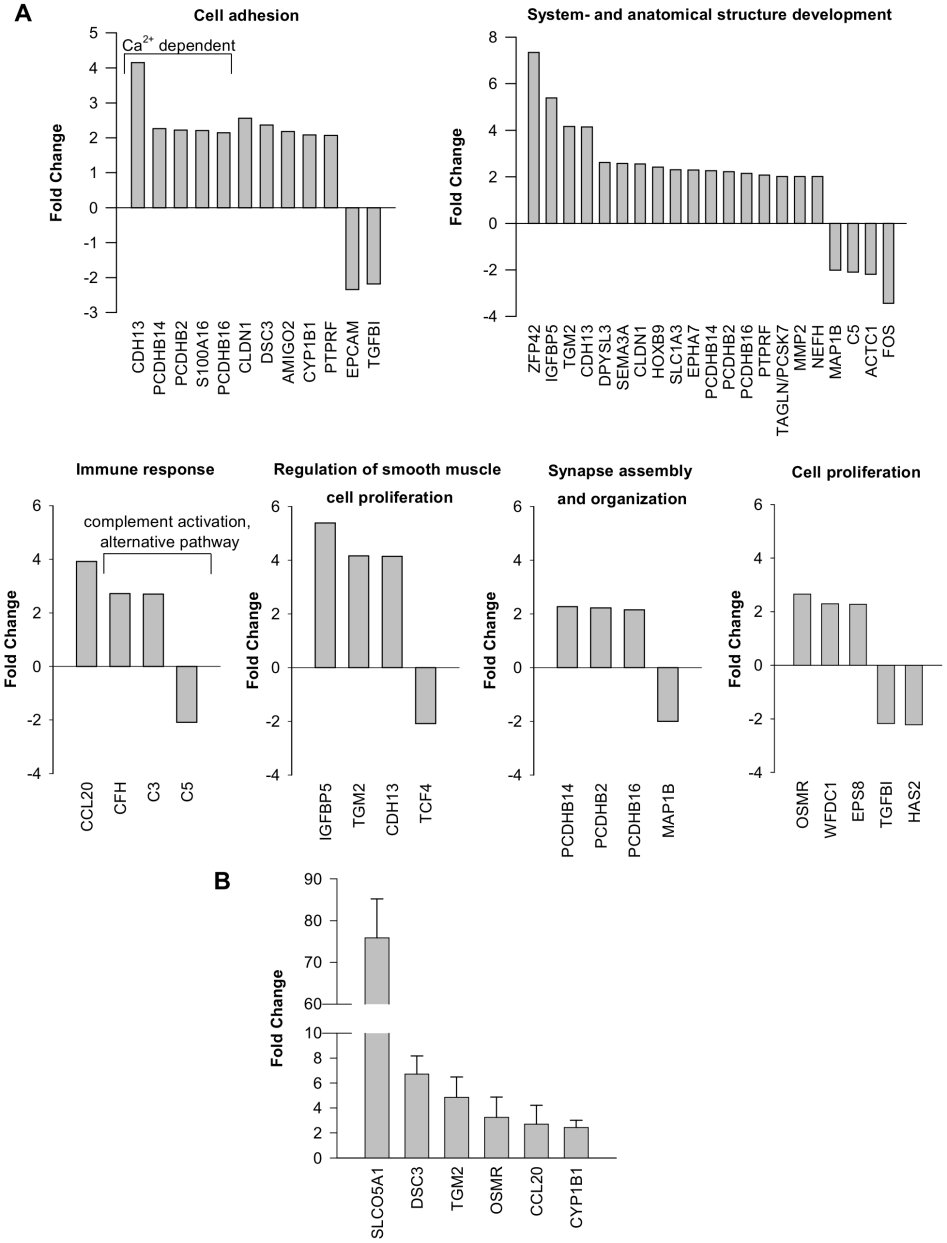
Analysis of gene expression using exon expression array and qRT-PCR. A) Gene expression profiling using exon expression arrays. RNA samples of mock-transfected HeLa cells respectively HeLa cells expressing the WT SLCO5A1 both treated with 1 µg/ml tetracycline for 24 h were collected and analyzed on Affymetrix Exon Arrays. Results of the WT SLCO5A1 sample were compared to the mock sample and expression values of genes with a fold-change of at least 2.0 were analyzed using the GeneSpring® GX 12.0 software. Selected genes were clustered according to their biological function using the GeneSpring Gene Ontology (GO) analysis tool (for complete results see supplemental information – Table S1). A fold-change expression of 30.2-fold was observed for SLCO5A1 (control) (not shown). B) Analysis of GeneChip Human Exon 1.0 ST microarray data by quantitative *real-time* PCR. The expression of the indicated genes was analyzed after application of mock-transfected HeLa cells and HeLa cells expressing the WT SLCO5A1 with tetracycline for 24 h. The relative expression levels of the WT SLCO5A1 sample were compared to the mock sample ( = 1) and normalized to GUSB (glucuronidase, beta) expression. Mean values with standard deviation of 3 biological replicates are displayed.

To further analyze whether the expression of SLCO5A1 alone alters the transcriptome of HeLa cells, an exon gene expression assay was carried out where mock-transfected HeLa cells were compared to WT SLCO5A1-expressing HeLa cells both treated with tet for 24 h. Genes regulated with a fold change of at least 2.0 were analyzed. Using the GeneSpring Gene Ontology (GO) analysis tool, the identified genes were categorized according to their biological function (Fig. 5A). The majority of genes are implicated in the processes ‘system development’ (GO:0048731), ‘anatomical structure development’ (GO:0048856) (progression of an organismal system (groups of organs or tissues) over time, from its formation to the mature structure and progression of an anatomical structure from an initial condition to its mature state, respectively) (e.g. FBJ murine osteosarcoma viral oncogene homolog (FOS), transglutaminase 2 (TGM2)), and ‘cell-cell adhesion’ (GO:0016337) (e.g. desmocollin 3 (DSC3)). Furthermore, genes were identified which are involved in the biological processes ‘complement activation, alternative pathway’ (GO:0006957) (e.g. the complement components 3 and 5 (C3/5)), ‘regulation of smooth muscle cell proliferation’ (GO:0048660) (e.g. cadherin (heart) 13 (CDH13), transcription factor 4 (TCF4)), ‘synapse organization’ (GO:0050808), and ‘synapse assembly’ (GO:0007416) (e.g. microtubule associated protein 1B (MAP1B), protocadherin beta protein 2/14/16 (PCDHB2/14/16)). According to ‘Pub Med Gene’, the genes oncostatin M receptor (OSMR), hyaluronan synthase 2 (HAS2), epidermal growth factor receptor pathway substrate 8 (EPS8), WAP four-disulfide core domain 1 (WFDC1), and transforming growth factor, beta-induced (TGFBI) have been implicated in cell proliferation. The complete results of this microarray experiment can be found as supplemental information (Table S1).

The mRNA expression of the genes DSC3, TGM2, OSMR, and CCL20 (chemokine ligand 20) was found significantly upregulated in WT SLCO5A1-expressing HeLa cells compared to mock-transfected HeLa cells both stimulated with tet using qRT-PCR (Fig. 5B, S2). SLCO5A1 expression served as positive control (∼75-fold) (Fig. 5B). However, figure S2 also shows a higher expression of the genes DSC3, TGM2, OSMR, and CCL20 in WT SLCO5A1-expressing HeLa cells which were not stimulated with tet compared to mock-transfected HeLa cells (−/+ tet). We assume that this is due to the ‘leaky’ expression of SLCO5A1, but we can't exclude the possibility that the ‘SLCO5A1 expression effect’ is caused by an intrinsic cell line heterogeneity.

## Discussion

Although the SLCO5A1 protein has been detected in some cancerous tissues, it has not yet been biochemically and functionally well characterized. Here, we investigated the synthesis, assembly and post-translational processing of the human SLCO5A1 protein expressed in *X. laevis* oocytes and stably transfected HeLa cells. We found that the WT SLCO5A1 protein and its L^33^F mutant were readily synthesized in both cell types as membrane-bound proteins with at least three and potentially up to six N-glycans. The N-glycans were efficiently processed to the complex-type, indicating egress from the ER. In the *X. laevis* oocytes, both the core-glycosylated and the complex-glycosylated form of the SLCO5A1 protein appeared at the cell surface, although in distinct oligomeric states, homodimers and monomers, respectively. We suggest that the dimerization and persistence in the core-glycosylated state are related in a way that dimerization sterically hinders the processing of the core N-glycans to complex-type N-glycans during the transport through the Golgi apparatus.

Based on the same approach, i.e. expression in *X. laevis* oocytes and BN-PAGE, we have previously shown that within the SLC superfamily a conserved dimeric quaternary structure is inherent to the members of the SLC26 family including the motor protein prestin [34]. Prestin was first found by perfluoro-octanoate PAGE [35] and later by single particle analysis to assemble as a tetramer [36]. However, a recent low resolution structure of a bacterial SLC26 transporter corroborated a dimeric stoichiometry consistent with our data [37].

In HeLa cells we found that the WT SLCO5A1 and its L^33^F mutant were also expressed as complex-glycosylated and, to a lesser extent, as core-glycosylated proteins. Recently, Yao et al. (2012) identified the three glycosylation sites Asn134, Asn503 and Asn516 to participate in the glycosylation process of human OATP1B1 [38]. A simultaneous mutation of all three asparagines to glutamines resulted in a significantly reduced total protein amount as well as a loss of transport activity. Furthermore, it is assumed that posttranslational modification by N-glycosylation is important for OATPs because it seems that this modification is responsible for both their functional activity and delivery to the cell membrane [39].

Confocal fluorescence microscopy of the YFP-tagged SLCO5A1 revealed both an expression of SLCO5A1 protein in intracellular membranes and on the cell surface of HeLa cells. Although OATPs are usually expressed on the plasma membrane, strong intracellular staining was seen for both OATP1A2 in breast cancer and OATP1B3 in colon cancer [14]. Only partial expression of the SLCO5A1 protein on the plasma membrane of HeLa cells could result from aberrant posttranslational regulation such as phosphorylation, which for instance regulates the cell surface expression of human OATP2B1 [40].

The natural variant L^33^F of SLCO5A1 in comparison to WT SLCO5A1 showed no differences regarding its biochemical properties. Nevertheless, variations in genes encoding uptake transporters can alter expression in cells and can cause interindividual variations of drug effects [41].

Transport assays performed in *X. laevis* oocytes could not identify any of the substances tested as substrate for the WT SLCO5A1 protein or its L^33^F mutant. Although the SLCO5A1 protein was expressed on the cell surface of oocytes, it is unclear if the protein was functionally active. Perhaps the SLCO5A1 protein needs to be activated or transports through an unidentified exchange transport mechanism or only transports a still unidentified specific substrate; though SLCO5A1 seems to be widely expressed in many tissues. OATP2B1 and OATP2A1 were identified in *X. laevis* oocytes and could influence the uptake of potential OATP substrates [3]. Several lines of evidence suggest a crucial role of the large extracellular loop region 5, located between helix nine and ten, in the transport activity of OATPs [42]. A homology between this region and the Kazal-1/2-type serine protease inhibitors has been detected, which could be important for substrate binding [3], [43]. A typical Kazal domain contains six cysteine residues leading to three disulfide bonds with a 1-5/2-4/3-6 pattern [44]. Ten cysteine residues for the SLCO5A1 protein were predicted to be located in the extracellular loop region 5 by the membrane topology program MEMSAT 3. Metabolic pathways involving proteins with a Kazal-domain include the complement and coagulation cascades, the ECM-receptor interaction pathway and the TGF-beta signaling pathway [45], [46]. OATPs are the first example of this structure found as a domain in an integral membrane protein but the precise function of this domain is still unknown [3].

The transcriptional expression of SLCO transporters SLCO1A2, SLCO1B1, SLCO2B1, SLCO3A4 and SLCO4A1 in primary human antigen-presenting cells (APCs) was first described by Skazik et al. (2008) [9]. SLCO2B1, SLCO3A4 and SLCO4A1 are differentially expressed in monocytes, monocyte-derived macrophages and monocyte-derived mature dendritic cells. Interestingly, Skazik et al. (2008) observed that SLCO4A1 is highly expressed in macrophages compared to monocytes [9]. On the other hand, mature dendritic cells showed a lower SLCO4A1 expression compared to monocytes. Here we studied the transcriptional expression of SLCO5A1 in human primary blood cells. Data showed that SLCO5A1 expression decreases during the differentiation from monocytes to macrophages but increases during the differentiation from monocytes to mature dendritic cells. These results suggest that the differential expression of SLCO transporters might play a role in the differentiation process of APCs.

The effect of SLCO5A1 expression on global gene expression was analyzed in HeLa cells using exon expression arrays. Genes were identified that are implicated in the biological processes of cell-cell adhesion, system- and anatomical structure development, immune response, regulation of smooth muscle cell proliferation, and synapse assembly and -organization.

The calcium-dependent glycoprotein desmocollin 3 (DSC3), a member of the cadherin superfamily, is required for cell adhesion and desmosome formation/protein stabilization [47]. Transglutaminase 2 (TGM2), which is induced by retinoic acid, calcium-dependently catalyzes the crosslinking of proteins by epsilon-gamma glutamyl lysine isopeptide bonds. TGM2 colocalizes with extracellular matrix proteins [48], [49] and has been implicated in inflammatory responses [50]. The gene oncostatin M receptor (OSMR) encodes a member of the type I cytokine receptor family which is necessary for the response to oncostatin M (OSM) or IL-31. The OSMR is involved in the positive regulation of acute inflammatory responses and in cell proliferation. OSM induces dendritic cell maturation [51], and was also found to regulate drug transporter expression in human hepatocytes [52]. The chemokine CCL20 is mainly known because of its chemotactic activity. It represents a key factor for the recruitment of DCs into epithelial tissues [53], but also functions as an antimicrobial peptide in innate immunity [54].

DC maturation, migration, and formation of immunological synapses with T cells represent processes which depend on calcium mobilization and cytoskeletal reorganization [55]. Complex signaling cascades involving chemokines and cytokines regulate the DC immune response. Membrane transport proteins play a pivotal role in these signaling processes. Based on our findings, we hypothesize that SLCO5A1 might be involved in biological processes that are dependent on cell shape changes.

## Conclusion

Only very little is known about the organic anion transporting polypeptide OATP5A1. This study showed for the first time that human OATP5A1 is expressed as a N-complex-glycosylated polypeptide monomer or high-mannose-glycosylated homodimer that is localized in intracellular membrane compartments and on the plasma membrane. Furthermore, transcriptional analysis of SLCO5A1 and functional studies provided evidence that OATP5A1 might be a non-classical OATP family member which is involved in biological processes that require the reorganization of the cell shape, such as differentiation and migration. This finding would help to explain the increased expression of SLCO5A1/OATP5A1 in many cancerous tissues and cells throughout the body. But the substrate for the OATP5A1-mediated transport is still unidentified. Thus, further studies are required regarding the identification of substrates for OATP5A1 and the clarification of OATP5A1 biological function.

## Supporting Information

Figure S1 **alamarBlue® proliferation assay of hSLCO5A1-expressing HeLa cells.** 6×10^3^ cells were seeded in 12-wells with 1.5 ml medium in the absence or presence of 1 µg/ml tetracycline (tet). After 0 h, 24 h, 48 h, 72 h and 96 h alamarBlue® (AbD Serotec, Oxford, UK) in an amount equal to 10% of the volume in the well was added. Proliferation was measured using spectophotometry after a) 2 h and b) 4 h at 570 nm and 600 nm (*N* = 4). The percentage reduction of alamarBlue® was calculated with the mean values of the samples according to the equation 1 in the alamarBlue® Technical Datasheet.
(TIF)

Figure S2 **Analysis of GeneChip Human Exon 1.0 ST microarray data by quantitative** ***real-time*** **PCR.** The expression of the indicated genes was analyzed using mock-transfected HeLa cells and HeLa cells expressing the WT SLCO5A1 cultivated in the absence or presence of 1 µg/ml tetracycline (tet) for 24 h. The relative expression levels of the mock sample (+ tet) and the WT SLCO5A1 samples (−/+ tet) were compared to the mock sample (- tet) ( = 1) and normalized to GUSB (glucuronidase, beta) expression. Mean values with standard deviation of 3 biological replicates are displayed.
(TIF)

Table S1 **Analysis of gene expression of HeLa cells stably expressing hSLCO5A1 compared to mock-transfected cells using the Affymetrix GeneChip Human Exon 1.0 ST array.**
(PDF)
