# Supplementary figures and images for: Characterization of SLCO5A1/OATP5A1, a Solute Carrier Transport Protein with Non-Classical Function
Source: PLoS One. 2013 Dec 20;8(12):e83257. doi: 10.1371/journal.pone.0083257 (PMC3869781; doi:10.1371/journal.pone.0083257)

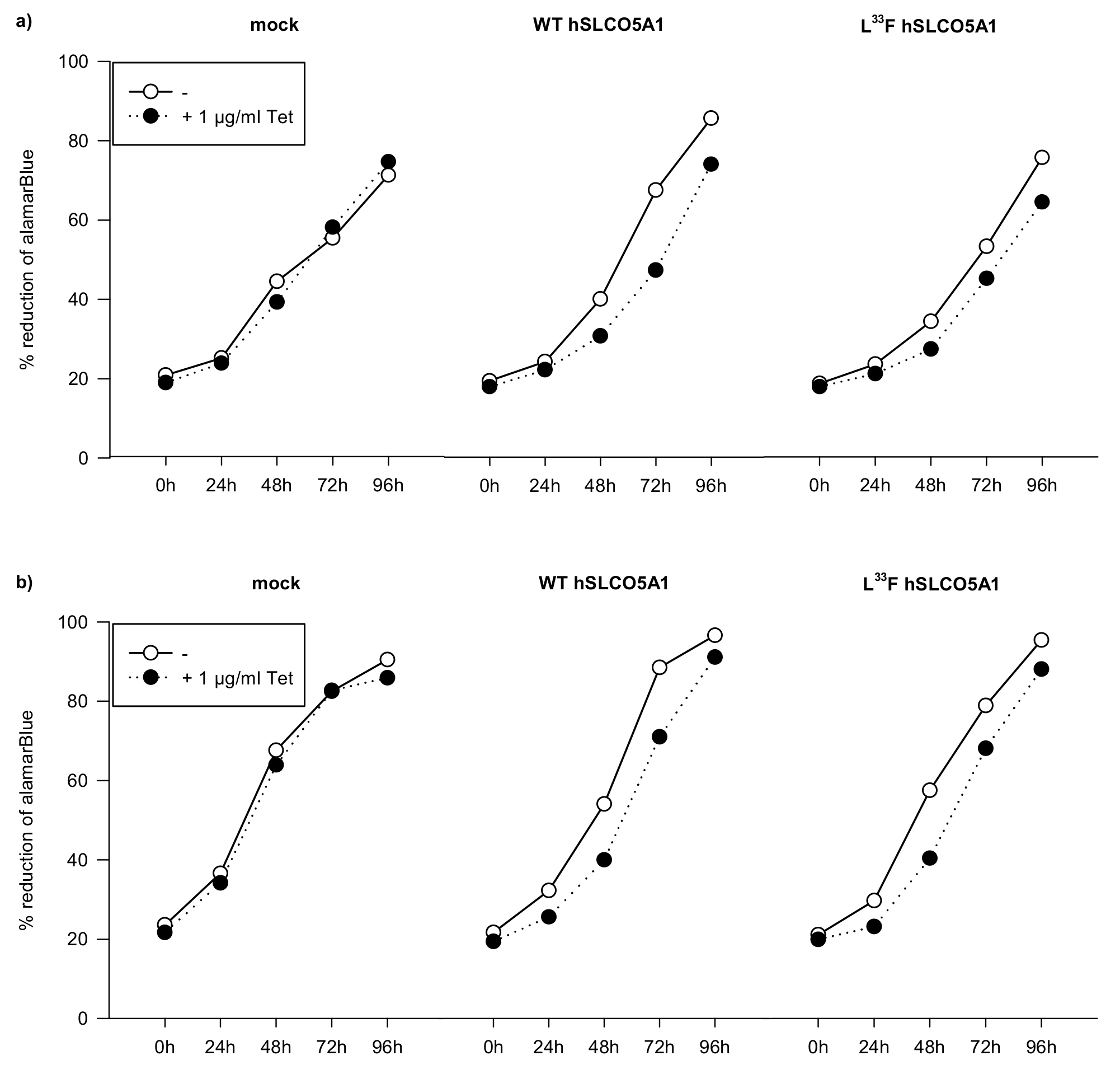

Supplement: Figure S1 — alamarBlue® proliferation assay of hSLCO5A1-expressing HeLa cells. 6×10^3^ cells were seeded in 12-wells with 1.5 ml medium in the absence or presence of 1 µg/ml tetracycline (tet). After 0 h, 24 h, 48 h, 72 h and 96 h alamarBlue® (AbD Serotec, Oxford, UK) in an amount equal to 10% of the volume in the well was added. Proliferation was measured using spectophotometry after a) 2 h and b) 4 h at 570 nm and 600 nm (N = 4). The percentage reduction of alamarBlue® was calculated with the mean values of the samples according to the equation 1 in the alamarBlue® Technical Datasheet. (TIF) [file pone.0083257.s001.tif]

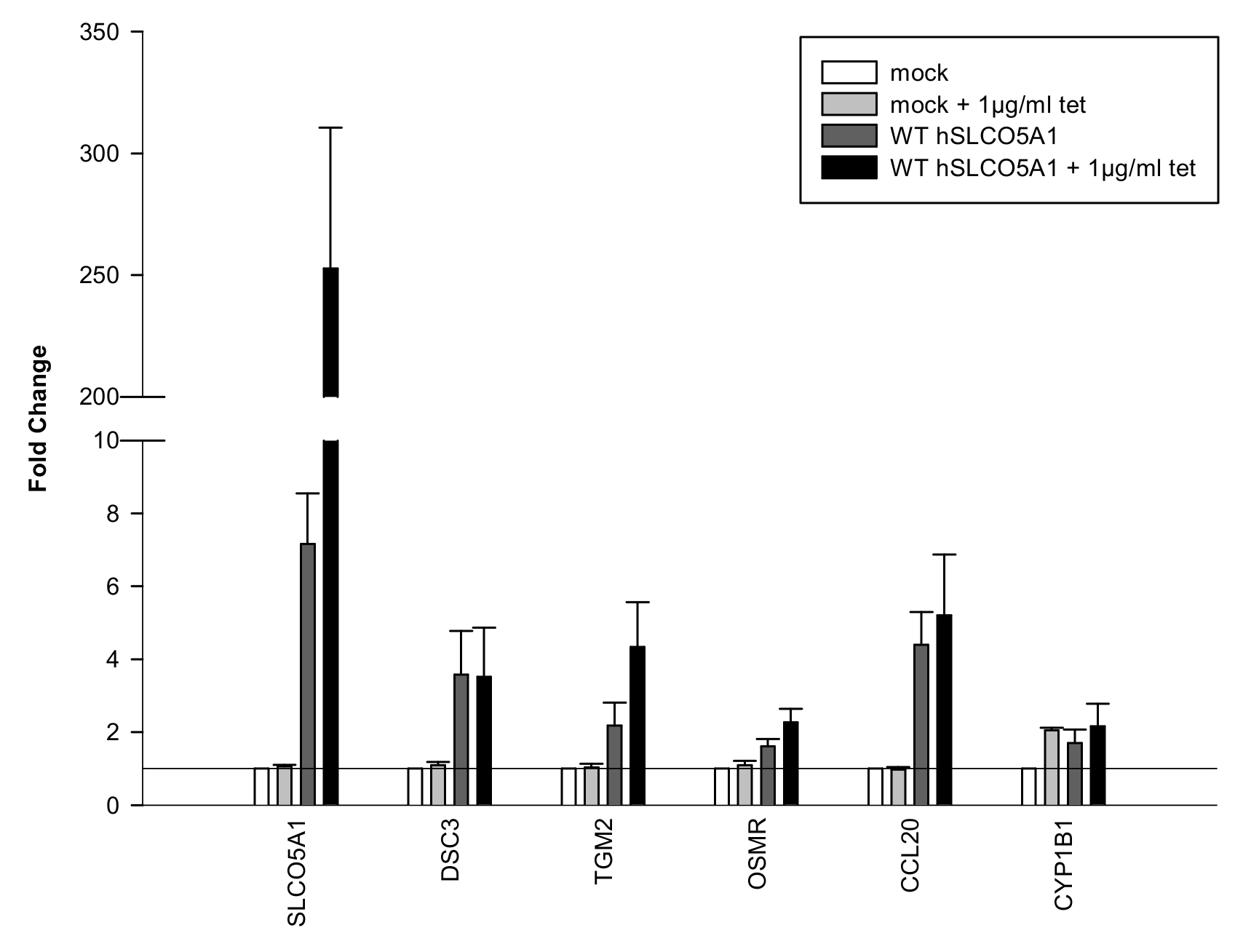

Supplement: Figure S2 — Analysis of GeneChip Human Exon 1.0 ST microarray data by quantitative real-time PCR. The expression of the indicated genes was analyzed using mock-transfected HeLa cells and HeLa cells expressing the WT SLCO5A1 cultivated in the absence or presence of 1 µg/ml tetracycline (tet) for 24 h. The relative expression levels of the mock sample (+ tet) and the WT SLCO5A1 samples (−/+ tet) were compared to the mock sample (- tet) ( = 1) and normalized to GUSB (glucuronidase, beta) expression. Mean values with standard deviation of 3 biological replicates are displayed. (TIF) [file pone.0083257.s002.tif]
