# Supplementary material for: Characterization of SLCO5A1/OATP5A1, a Solute Carrier Transport Protein with Non-Classical Function
Source: PLoS One. 2013 Dec 20;8(12):e83257. doi: 10.1371/journal.pone.0083257 (PMC3869781; doi:10.1371/journal.pone.0083257)
Supplement: Table S1 — Analysis of gene expression of HeLa cells stably expressing hSLCO5A1 compared to mock-transfected cells using the Affymetrix GeneChip Human Exon 1.0 ST array. (PDF) [file pone.0083257.s003.pdf]

Table S1: Analysis of gene expression of Hela cells stably expressing hSLCO5A1 compared to mock-transfected cells using the Affymetrix GeneChip Human Exon 1.0 ST array.

**Transcript Cluster ID**	**FC [SLCO5A1] vs [mock]**	**Reg.**	**Gene description**	**Gene symbol**	**Refseq.**
8151223	30,211792	up	solute carrier organic anion transporter family, member 5A1	SLCO5A1	NM_030958
8098690	7,344609	up	zinc finger protein 42 homolog (mouse)	ZFP42	NM_174900
8090180	6,3791203	up	mucin 13, cell surface associated	MUC13	NM_033049
8058857	5,3928714	up	insulin-like growth factor binding protein 5	IGFBP5	NM_000599
7911231	4,8719125	up	olfactory receptor, family 2, subfamily W, member 3	OR2W3	NM_001001957
7911229	4,868381	up	olfactory receptor, family 2, subfamily W, member 3	OR2W3	NM_001001957
7930181	4,764688	up	arsenic (+3 oxidation state) methyltransferase | chromosome 10 open reading frame 32	AS3MT|C10orf32	NM_020682
7911218	4,527852	up	tripartite motif-containing 58 | olfactory receptor, family 2, subfamily W, member 3	TRIM58|OR2W3	NM_015431
8025992	4,4089227	up	zinc finger family member 788	ZNF788	NR_027049
8066214	4,158959	up	transglutaminase 2 (C polypeptide, protein-glutamine-gamma-glutamyl-transferase)	TGM2	NM_004613
7997504	4,145767	up	cadherin 13, H-cadherin (heart)	CDH13	NM_001257
8048864	3,9234626	up	chemokine (C-C motif) ligand 20	CCL20	NM_004591
8166611	3,3135734	up	melanoma antigen family B, 2	MAGEB2	NM_002364
7985147	2,8416505	up	DnaJ (Hsp40) homolog, subfamily A, member 4	DNAJA4	NM_018602
7908459	2,723075	up	complement factor H	CFH	NM_000186
8033257	2,6953452	up	complement component 3	C3	NM_000064
8105040	2,6588984	up	oncostatin M receptor	OSMR	NM_003999
8114920	2,6222978	up	dihydropyrimidinase-like 3	DPYSL3	NM_001197294
8135943	2,619421	up			
8140668	2,5663838	up	sema domain, immunoglobulin domain (Ig), short basic domain, secreted, (semaphorin) 3A	SEMA3A	NM_006080
8092726	2,5568209	up	claudin 1	CLDN1	NM_021101
7957260	2,5054543	up	GLI pathogenesis-related 1 | KRR1, small subunit (SSU) processome component, homolog (yeast)	GLIPR1|KRR1	NM_006851
8016476	2,4192357	up	homeobox B9	HOXB9	NM_024017
8022692	2,3732793	up	desmocollin 3	DSC3	NM_024423
7962058	2,369142	up	transmembrane and tetratricopeptide repeat containing 1	TMTC1	NM_175861
8104930	2,3014822	up	solute carrier family 1 (glial high affinity glutamate transporter), member 3	SLC1A3	NM_004172

7997582	2,2981057	up	WAP four-disulfide core domain 1	WFDC1	NM_021197
8128284	2,2859817	up	EPH receptor A7	EPHA7	NM_004440
8100808	2,2834702	up	sulfotransferase family 1E, estrogen-preferring, member 1	SULT1E1	NM_005420
7961546	2,2785645	up	epidermal growth factor receptor pathway substrate 8	EPS8	NM_004447
8117301	2,2755892	up	histone cluster 1, H2ba	HIST1H2BA	NM_170610
8108744	2,2554677	up	protocadherin beta 14	PCDHB14	NM_018934
8108683	2,2208428	up	protocadherin beta 2	PCDHB2	NM_018936
7920291	2,214168	up	S100 calcium binding protein A16	S100A16	NM_080388
7950906	2,1988966	up	cathepsin C	CTSC	NM_001814
7962579	2,1802745	up	adhesion molecule with Ig-like domain 2	AMIGO2	NM_001143668
7974835	2,1631925	up	protein kinase C, eta	PRKCH	NM_006255
7958842	2,1588798	up			
8108716	2,1456244	up	protocadherin beta 16	PCDHB16	NM_020957
8051583	2,0751958	up	cytochrome P450, family 1, subfamily B, polypeptide 1	CYP1B1	NM_000104
7900792	2,0665143	up	protein tyrosine phosphatase, receptor type, F	PTPRF	NM_002840
8072382	2,0590732	up	oxysterol binding protein 2	OSBP2	NM_030758
8046815	2,048808	up	zinc finger protein 804A	ZNF804A	NM_194250
7992861	2,0247858	up			
8154233	2,0219495	up		CD274	NM_014143
7944082	2,014723	up	transgelin | proprotein convertase subtilisin/kexin type 7	TAGLN|PCSK7	NM_001001522
7956269	2,0133724	up			
8072229	2,0132298	up	neurofilament, heavy polypeptide	NEFH	NM_021076
8092541	2,0126252	up	lipase, member H	LIPH	NM_139248
7995681	2,0097687	up	matrix metallopeptidase 2 (gelatinase A, 72kDa gelatinase, 72kDa type IV collagenase)	MMP2	NM_004530
8149248	2,00845	down			
8106098	2,010132	down	microtubule-associated protein 1B	MAP1B	NM_005909
7911854	2,0689807	down		KIAA0495	NR_033711
8023415	2,088585	down	transcription factor 4	TCF4	NM_001083962
8163839	2,0930207	down	complement component 5	C5	NM_001735
7950162	2,0998118	down	phosphodiesterase 2A, cGMP-stimulated	PDE2A	NM_002599
7997642	2,108236	down	cysteine-rich secretory protein LCCL domain containing 2	CRISPLD2	NM_031476
8114898	2,1371043	down			
8055314	2,169951	down	LY6/PLAUR domain containing 1 | G protein-coupled receptor 39	LYPD1|GPR39	NM_144586
8108217	2,1811423	down	transforming growth factor, beta-induced, 68kDa	TGFBI	NM_000358
7987315	2,1928756	down	actin, alpha, cardiac muscle 1	ACTC1	NM_005159

7959330	2,196594	down	WD repeat domain 66	WDR66	NM_144668
8152617	2,2164152	down	hyaluronan synthase 2	HAS2	NM_005328
7981514	2,2531073	down	AHNAK nucleoprotein 2	AHNAK2	NM_138420
8114900	2,2627628	down	protein phosphatase 2, regulatory subunit B, beta	PPP2R2B	NM_004576
8067652	2,29772	down	eukaryotic translation elongation factor 1 alpha 2	EEF1A2	NM_001958
8157231	2,3248549	down			
8098439	2,341663	down	epithelial cell adhesion molecule	EPCAM	NM_002354
8004152	2,3520849	down	WSC domain containing 1	WSCD1	NM_015253
7970793	2,4908414	down	solute carrier family 46, member 3	SLC46A3	NM_181785
8131844	2,7798915	down	glycoprotein (transmembrane) nmb	GPNMB	NM_001005340
7942332	3,0415804	down	folate receptor 1 (adult)	FOLR1	NM_016724
8171248	3,3562174	down	Kallmann syndrome 1 sequence	KAL1	NM_000216
7975779	3,4330106	down	FBJ murine osteosarcoma viral oncogene homolog	FOS	NM_005252
